# Does a maternal history of abuse before pregnancy affect pregnancy outcomes? A systematic review with meta-analysis

**DOI:** 10.1186/s12884-018-2030-8

**Published:** 2018-10-16

**Authors:** Maryam Nesari, Joanne K Olson, Ben Vandermeer, Linda Slater, David M Olson

**Affiliations:** 1 Faculty of Nursing, University of Alberta, Edmonton, AB Canada; 2 Alberta Research Centre for Health Evidence, Department of Pediatrics, University of Alberta, Edmonton, AB Canada; 3 John W. Scott Health Sciences Library, University of Alberta, Edmonton, AB Canada; 4 Departments of Obstetrics and Gynecology, Pediatrics and Physiology, University of Alberta, Edmonton, AB T6G 2S2 Canada

**Keywords:** Maternal, Abuse before pregnancy, Preterm delivery, Low birth weight

## Abstract

**Background:**

Evidence relating maternal history of abuse before pregnancy with pregnancy outcomes is controversial. This study aims to examine the association between maternal histories of abuse before pregnancy and the risk of preterm delivery and low birth weight.

**Methods:**

We searched Subject Headings and keywords for exposure and the outcomes through MEDLINE, EMBASE, Cochrane Database of Systematic Reviews, Cochrane Central Register of Controlled Trials, Psycinfo, CINAHL, Scopus, PILOTS, ProQuest Dissertations & Theses Global and Web of Science Core Collection in April 2017. We selected original studies that reported associations between maternal histories of abuse of any type and either preterm delivery or low birth weight. Studies that included interventions during pregnancy to lower maternal stress but reported no control data were excluded. We utilized the Newcastle-Ottawa Quality Assessment Scales for observational studies to assess the risk of bias in the primary studies. Two independent reviewers performed the selection of pertinent studies, assessment of risk of bias, and data extraction. Unadjusted pooled odds ratios (OR) with 95% Confidence Interval (CI) were calculated for the two outcomes of preterm delivery and low birth weight in 16 included studies.

**Results:**

Maternal history of abuse before pregnancy was significantly associated with preterm delivery (OR 1.28, 95% CI: 1.12–1.47) and low birth weight (OR 1.35, 95% CI: 1.14–1.59). A substantial level of heterogeneity was detected within the two groups of studies reporting preterm birth and low birth weight (I^2^ = 75% and 69% respectively). Subgroup analysis based on the specific time of abuse before pregnancy indicated that childhood abuse increases the risk of low birth weight by 57% (95% CI: 0.99–2.49). When the included studies were categorized based on study design, cohort studies showed the highest effect estimates on preterm delivery and low birth weight (OR: 1.69, 95%CI: 1.19–2.40, OR: 1.56, 95% CI: 1.06–2.3, respectively).

**Conclusions:**

We recommend that more high quality research studies on this topic are necessary to strengthen the inference. At the practice level, we suggest more attention in detecting maternal history of abuse before pregnancy during antenatal visits and using this information to inform risk assessment for adverse pregnancy outcomes.

**Trial registration:**

Registration number: PROSPERO (CRD42016033231).

**Electronic supplementary material:**

The online version of this article (10.1186/s12884-018-2030-8) contains supplementary material, which is available to authorized users.

## Background

Maternal chronic stress is increasingly recognized as a risk factor for some pregnancy outcomes such as Preterm Delivery (PTD) andLow Birth Weight (LBW). [1–3] PTD, the major perinatal health problem [4] is identified by the World Health Organization (WHO) as the primary cause of death in children less than five years old. [5] It is associated with impaired developmental trajectories [6, 7] and can lead to adult cardiovascular [8, 9] and metabolic diseases. [10–13] Infants born with LBW often experience severe health problems and developmental issues leading to substantial healthcare costs. [14]

In animal models, it is hypothesized that accumulated prenatal maternal stress leads to a high stress load that shortens gestation length or causes other adverse pregnancy and behavioral outcomes. [15, 16] However, if the stressed subject is placed into a supportive environment, many of the adverse effects of stress can be reversed, suggesting that stress is a modifiable risk factor. [17, 18] In human research, prenatal maternal stressors, such as natural disasters [19] adverse life events, and daily perceived stress [20] are associated with adverse pregnancy outcomes. Abuse and in particular, intimate partner violence (IPV), are the widely researched stressors associated with pregnancy outcomes. Abuse is defined as any attempt to control the behavior of another person and encompasses any direct or indirect physical, sexual or emotional maltreatment. [21] IPV refers to any maltreatment within an intimate relationship that leads to physical, psychological or sexual harm to those in the relationship.

Systematic reviews with meta-analyses indicated that IPV *during pregnancy* associates with PTD and low LBW. [21–24] Moreover, in some primary investigations, maternal history of abuse *before pregnancy* has been associated with adverse pregnancy outcomes; however others reported no association in this regard. [3, 25–33] Two recent systematic reviews examining the existing evidence on the relation between childhood sexual abuse and subsequent adult sequelae found that women who had experience of childhood abuse tended to have more problems and complaints during their pregnancy; [34, 35] nevertheless, both studies reported that the results of the associations between maternal history of childhood abuse and PTD and LBW were inconsistent. [34, 35] Therefore, this systematic review aims to examine whether maternal history of life-long abuse *before pregnancy* is associated with PTD and LBW.

## Objective

The purpose of this systematic review was to assess the association between maternal history of abuse (physical, emotional, and sexual) at any time in life *before pregnancy* and risk of PTD and LBW. This systematic review is unique as we classified the relevant literature based on time of abuse to have a clear understanding of the effect of the time of abuse on the outcomes of interest. Consequently, three subgroups of childhood abuse (abuse happened before 18 years of age), anytime abuse (history of abuse anytime during married life until 12 months before pregnancy), and recent abuse (abuse occurred during 12 months before pregnancy) were created.

## Methods

We followed the Meta-Analysis of Observational Studies in Epidemiology (MOOSE) criteria for reporting this meta-analysis.

### Information sources, search strategy, eligibility criteria

**Fig. 1:**
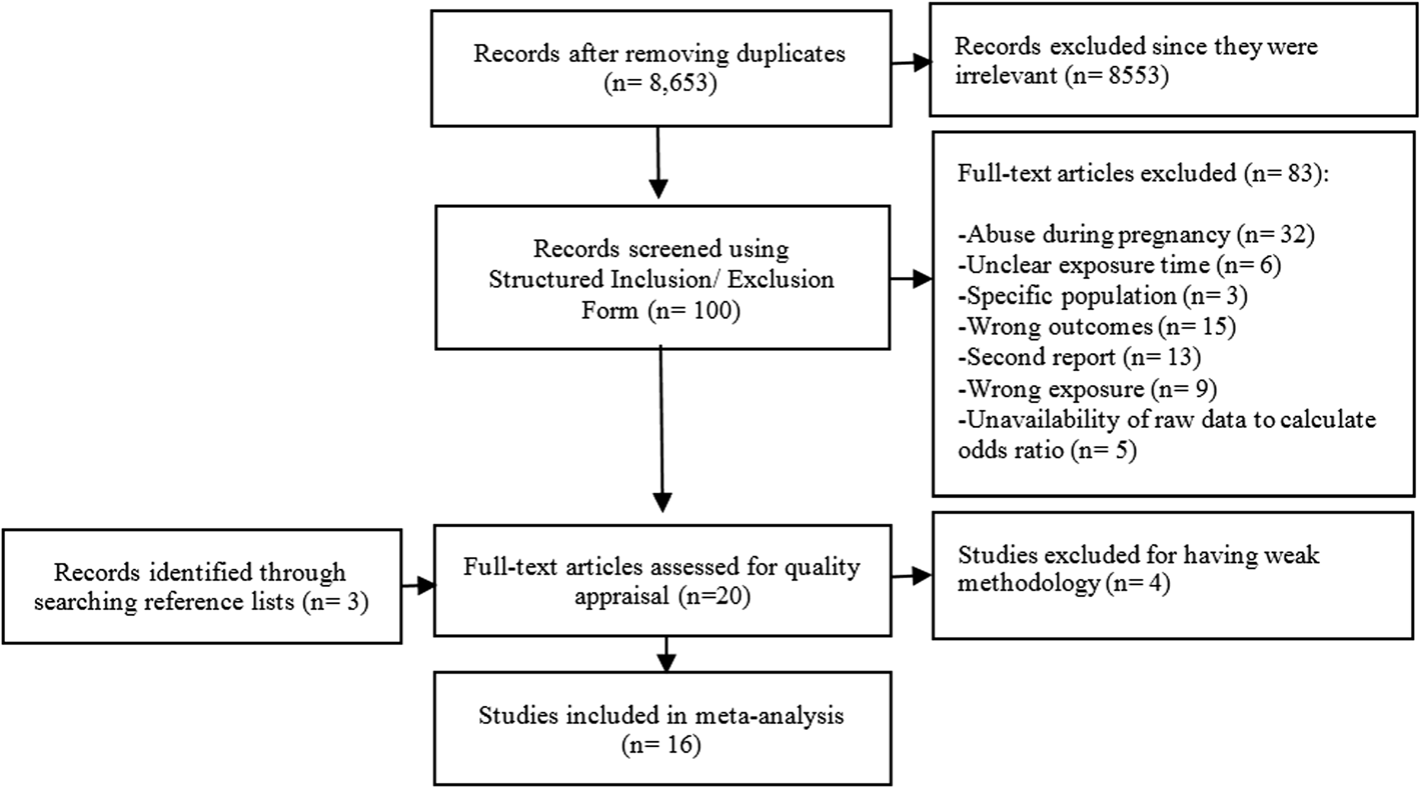
Flowchart of search results. The flowchart of search results and process for identification, selection and inclusion of the studies in the systematic review is shown

We conducted an initial search intended to scope the literature on the association between lifelong stressors (including but not limited to sexual, physical and emotional abuse) and PTD in July 2015. Databases searched were MEDLINE, EMBASE, Psycinfo, Scopus, Web of Science Core Collection, CINAHL, PILOTS, Cochrane Database of Systematic Reviews and Cochrane Central Register of Controlled Trials. The search strategy is presented in Additional file 1. We performed an updated, more focused, search of MEDLINE, EMBASE, Scopus, Web of Science Core Collection, CINAHL, PILOTS, Violence and Abuse Abstracts, ProQuest Dissertations & Theses Global and Cochrane Database of Systematic Reviews and Cochrane Central Register of Controlled Trials in April 2017 to retrieve literature that studied the association between sexual, physical and emotional abuse and PTD as well as LBW. Full strategies for this search are contained in Additional file 2. To ensure comprehensive coverage of the literature, we screened the results of both searches. Search results are available in Fig. 1.

We initially screened citations to identify potentially relevant studies. Then we retrieved full-texts of the selected articles and assessed them further for eligibility according to a structured inclusion/exclusion form used by the two reviewers. The two steps of the selection process were performed by two reviewers independently, apparent discrepancies between them were resolved by consensus. We selected original studies that reported associations between maternal history of abuse of any kind and either PTD or LBW. Studies that included pharmacological or psychosocial intervention during pregnancy to lower maternal stress but report no control data were excluded. When more than one report was published based on the same sample, we included the more comprehensive report in our analysis.

For this review, abuse is defined as an attempt to control the behavior of another person and encompasses any direct or indirect physical, sexual or emotional maltreatment at any age, before pregnancy. [21] The primary outcomes in this review are PTD, defined as giving birth to a singleton at less than 37 weeks of gestation and LBW, defined as a birth weight of a live born infant of less than 2500 g regardless of gestational age.

We searched reference lists in each of the included articles as well as relevant review articles manually. Subsequently, we also examined reference lists of the newly identified articles. Additionally, we e-mailed individual researchers or organizations working on birth outcomes and maternal experience of abuse and violence to determine whether any published or unpublished studies existed that were not retrieved by our search.

### Assessment of risk of bias

For the assessment of methodological quality (risk of bias) of the included studies, we utilized the Newcastle-Ottawa Quality Assessment Scales for observational studies. These scales are comprised of seven items that evaluate three domains of quality: sample selection, comparability of cohorts, and assessment of outcomes. A total score of 6 to 8 stars indicated high quality, 4 or 5 stars indicated moderate quality, and 3 or fewer stars related to poor quality. Two researchers from our team conducted the assessment of methodological quality of the included studies independently. Disagreement was discussed among the entire research team. We presented the results of the assessment in Table 1.

**Table 1:** Characteristics of studies included in the meta-analysis

First Author /Pub Date	Country	Participants	Study Design	Sample size	Type of Abuse	Time of Abuse	Outcomes	Study Quality
Christiaens/2015 [3]	Canada	All ethnic groups	Case- control	622	Childhood abuse	Childhood	PTD	7
Silverman/2006 [29]	USA	All ethnic groups	Case- control	118,579	Intimate partner physical Abuse	Recent	PTD LBW	7
Jagoe/ 2000 [37]	USA	Low risk nonurban population	Cohort	84	Intimate partner physical Abuse	Anytime in married life	PTD LBW	5
Campbell/1999 [27]	USA	All ethnic groups	Case-control	252	Intimate partner emotional physical, and sexual abuse	Anytime in married life	LBW	5
Grimstad/ 1999 & 1997 [25, 36]	Norway	All ethnic groups	Case-control	174	Sexual Abuse Intimate partner sexual abuse	Childhood Anytime in married life	PTD LBW	4
Neggers/ 2004 [28]	USA	Low income/low risk population(82% African-American)	Case-control	3103	Intimate partner physical abuse leading to Injury	Recent	PTD LBW	4
Stevens- Simon/1994 [39]	USA	Low income, African American	Cohort	127	Physical abuse	Childhood	PTD LBW	5
Noll/ 2007 [30]	USA	All ethnic groups	Cohort	186	Sexual abuse	Childhood	PTD	8
Henriksen/ 2014 [42]	Norway	All ethnic groups	Cohort	76,870	Sexual violence (severe, mild, moderate)	Childhood Anytime in married life Recent	PTD LBW	7
Taft/ 2007 [31]	Australia	All ethnic groups	Cohort	9692	Intimate partner violence	Anytime in married life	PTD	5
Leeners/ 2014 [33]	Switzerland	All ethnic groups	Cohort	255	Sexual abuse	Childhood	PTD	8
Curry/ 1998 [26]	USA	All ethnic groups	Cohort	1597	Physical abuse Sexual abuse	Recent	LBW	7
Fried / 2008 [41]	USA	All ethnic groups	Cross-sectional	1555	Emotional, physical, Sexual abuse	Anytime in married life	PTD LBW	8
Selk/2016 [51]	USA	Nurses	Case- control	51,434	Physical abuse Sexual abuse	Childhood	PTD	6
Harville/2010 [32]	UK	All ethnic group	Cohort	4865	Violence	Childhood	LBW PTD	6
Scribano/ 2013 [38]	USA	Low income mothers	Case- control	10,855	Intimate partner violence	Recent	LBW PTD	7

Characteristics of all studies that were included in the meta-analysis are presented

### Data extraction

We utilized a researcher-constructed data extraction sheet piloted with 10 studies to record data from the included studies. Two researchers from our team extracted the data independently; any inconsistencies between them were resolved by reviewing the full text articles.

### Data synthesis

We conducted a Meta-analysis using Review Manager 5.2 (Copenhagen: The Nordic Cochrane Centre, 2012). We calculated odds ratios (OR) with 95% confidence intervals (CI) for those included articles that did not report these measures. Weighting of the studies in this meta-analysis was calculated based on the inverse variance of the study. We assessed the statistical heterogeneity (variability among the studies’ results) using the I-squared statistic. We categorized the included studies into three categories according to the time of maternal abuse: childhood abuse, anytime abuse, recent abuse. Pooled OR were computed to estimate the association between maternal experience of abuse and PTD and LBW within each of the subgroups. Despite our expectation, some included articles reported only unadjusted OR for the outcomes variables, and there was not enough data available from these studies to calculate an adjusted OR for them. Also, adjusted confounders were varied among those studies that reported both adjusted and unadjusted OR for the outcome variables. Therefore, we decided to perform the meta-analysis of unadjusted data. We chose a random effects model that accounted for a degree of clinical and statistical heterogeneity that was expected among the included studies. We created funnel plots of the data to assess for the possibility of small study bias. Since all the included studies in the meta-analysis were from high-income countries (United States of America, United Kingdom, Canada, Switzerland, Norway, Australia), we did not conduct subgroup analysis based on study context.

## Results

### Study selection

After removing duplicates, 8653 records were retained in total. Out of this, we excluded 8553 articles during the titles and abstract review, and another 83 articles using the structured inclusion/exclusion form. We summarized the reasons for these exclusions in Fig. 1. Through manually searching the reference lists of the included articles, we added three additional studies. Finally, we emailed 13 researchers who may have had relevant manuscripts submitted or in press. Only one responded and indicated that she did not have a relevant article.

From 20 eligible studies, we excluded four because of weak methodologies. This resulted in 16 studies that were included in the meta-analysis. Some of these studies reported both PTD and LBW as outcome variables, while some reported either PTD or LBW. We divided the 16 studies into two groups based on the outcome variables they reported. We included two reports from one study. [25, 36] As both reports presented data on different outcomes from one sample, we treated them as one study.

### Study characteristics

We presented the characteristics of the included studies in Table 1. The majority (62.5%) of the studies originated from the United States and the remainder were from other high income countries. The study designs are cross-sectional, case-control and cohort studies that assessed the association between abuse and PTD or LBW. Sample size in these studies ranged from 84 to 118,579. These samples encompass non-abused women and women who had experienced abuse during their childhood, anytime during their married life, or during the 12 months before pregnancy.

The sample for the majority of these studies included people from all ethnic groups in the country where the study was conducted; however, only one study examined a low risk nonurban population [37] and three studies assessed low income populations. [28, 38, 39] Abuse for subjects in almost 45% of the included studies was defined as abuse that originated from an intimate partner. Measurement tools for abuse varied across the studies. Data related to maternal history of abuse in the included articles were obtained by self-report and were collected through interviews, self-administered questionnaires, or obtained from databases.

### Preterm delivery

**Fig. 2:**
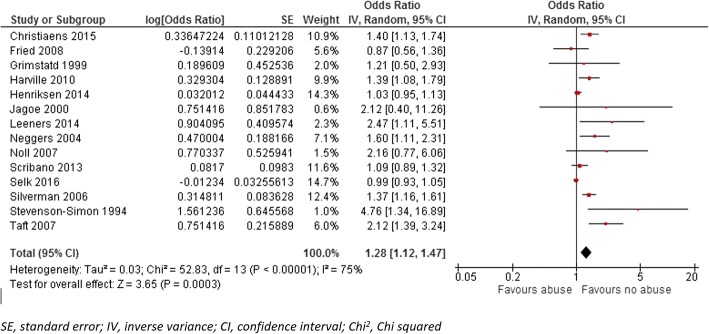
Forest plot of overall unadjusted effect estimate for preterm birth. The Forest plot of overall unadjusted effect estimate for preterm birth is shown

**Fig. 3:**
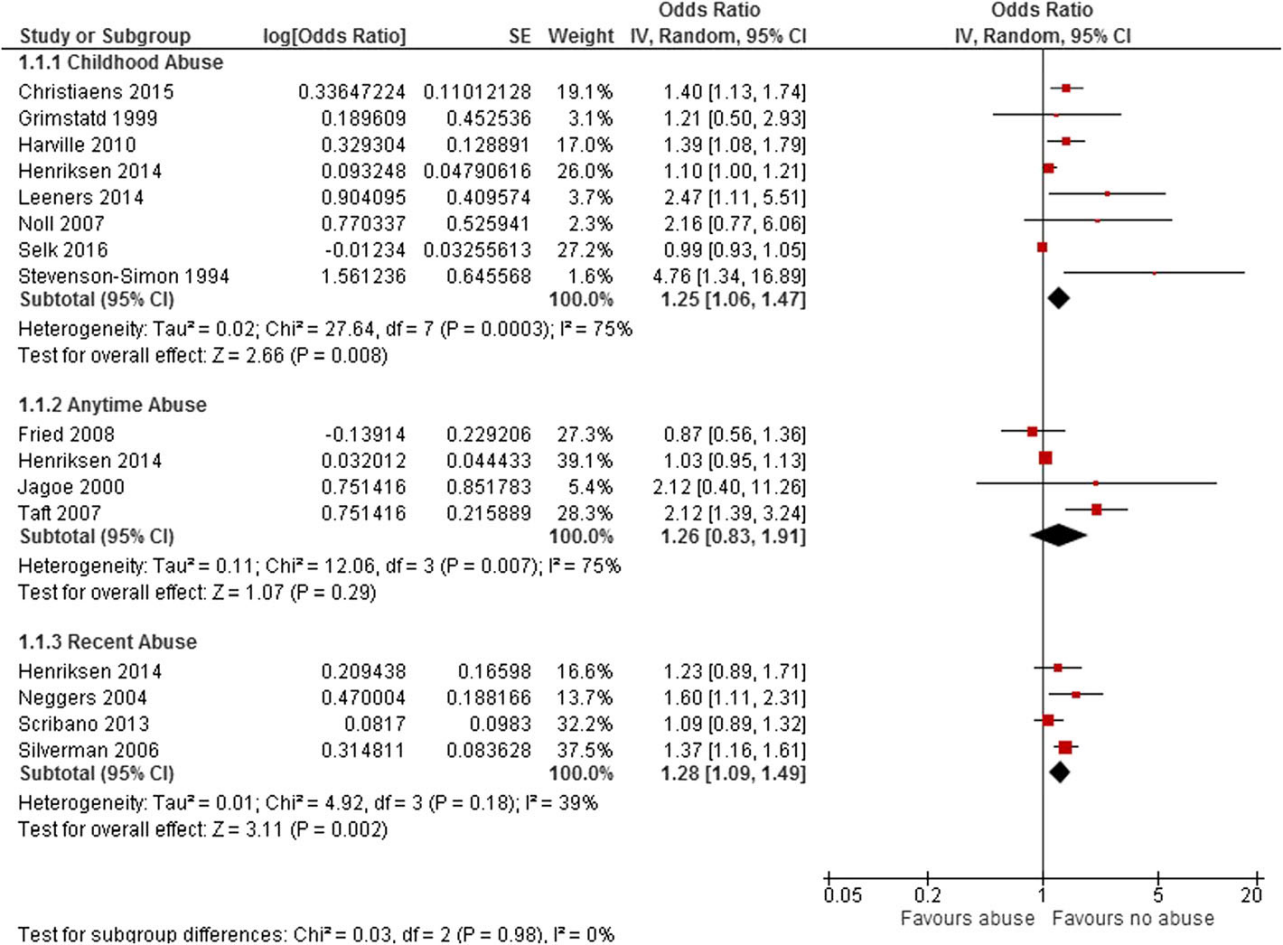
Forest plots of unadjusted effect estimate for preterm birth in subgroups of abuse time. The Forest plots of unadjusted effect estimate for preterm birth when studies categorized based on time of abuse are shown

Fourteen studies reported an association between maternal history of abuse in different points of life before pregnancy and PTD. Pooled OR for PTD among these 14 studies was 1.28 (95% CI: 1.12–1.47, *p* < 0.000); however, we detected a substantial level of heterogeneity among the studies in this group (I^2^ = 75%) (Fig. 2). When we conducted analysis within subgroups, having an experience of sexual abuse during childhood, reported by eight studies, increased the odds of PTD by 25% (OR 1.25, 95% CI: 1.06–1.47, *p* = 0.008) compared to women who did not have that experience. The odds of PTD were 26% higher (OR 1.26, 95% CI: 0.83–1.91, *p* = 0.29) in women who experienced sexual or physical abuse any time during their married life before pregnancy, (reported by four studies), compared to those who did not have such an experience. In addition, when maternal abuse happened within the 12 months before pregnancy (reported by four studies), the odds of PTD increased by 28% (OR 1.28, 95%CI: 1.09–1.49, *p* = 0.002) (Fig. 3). We detected a substantial level of heterogeneity among the studies in the anytime abuse and childhood abuse group (I^2^ = 75%). The funnel plot for all studies included in PTD analysis was asymmetric (Additional file 3). However, as the number of studies included is low (14 studies) the power of the plot to distinguish chance from real asymmetry is low. [40] Hence, we conducted an Egger test that indicated a likely publication bias (*P* < 0.003) among included articles reporting PTD.

**Fig. 4:**
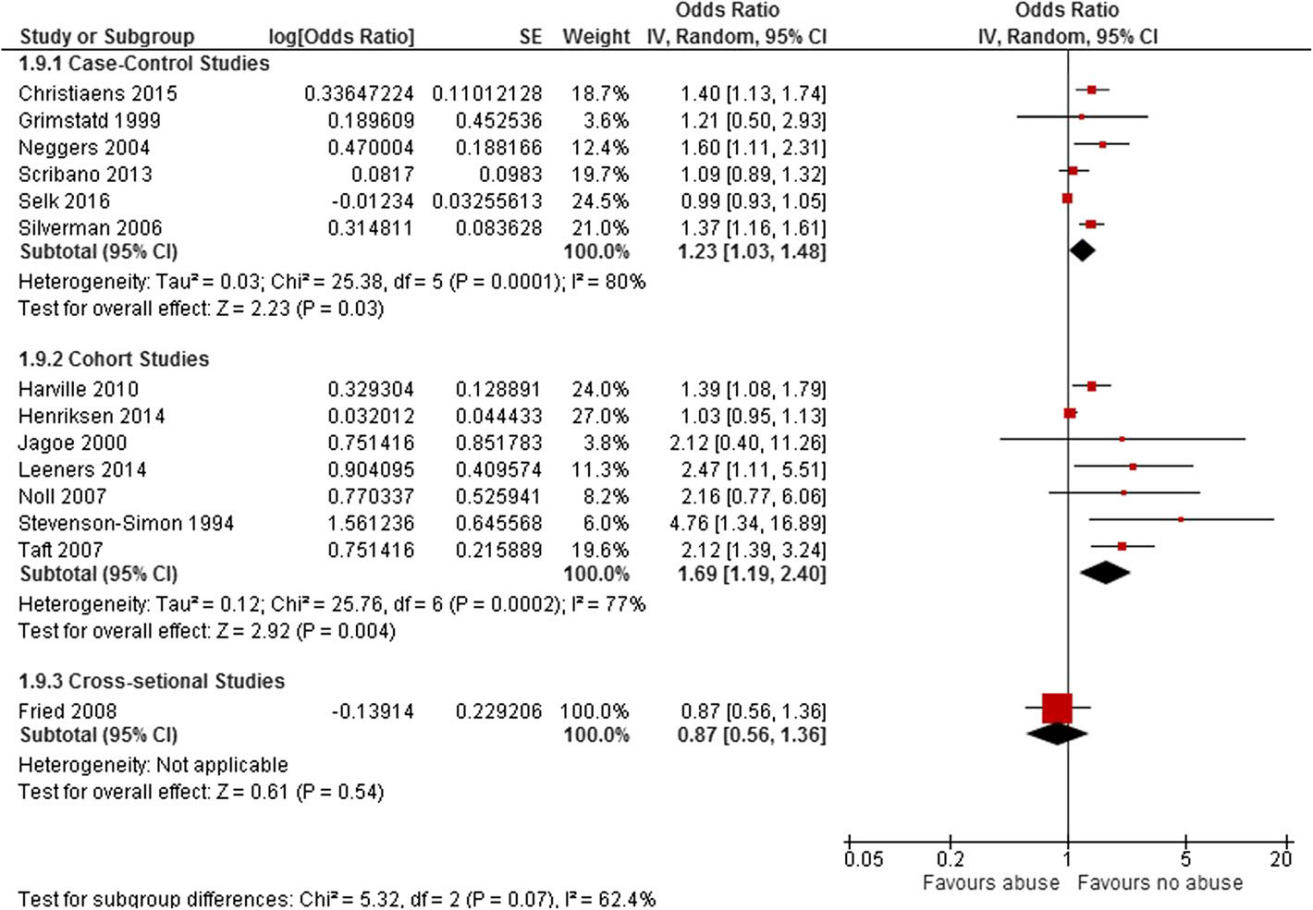
Forest plots of unadjusted effect estimate for preterm birth in subgroups of study design. The Forest plots of unadjusted effect estimate for preterm birth when studies categorized based on study design are shown

Furthermore, we stratified the studies by study design (cohort, case- control, and cross-sectional). The pooled OR for PTD  among seven studies with cohort designs was 1.69 (95%CI: 95%, 1.19–2.40, *p* = 0.004), which was higher than the effect estimated for six studies with case-control designs (OR: 1.23, 95% CI: 1.03–1.48, *p* < 0.03). There was only one study with a cross-sectional design in this group [41] (Fig. 4).

### Low birth weight

**Fig. 5:**
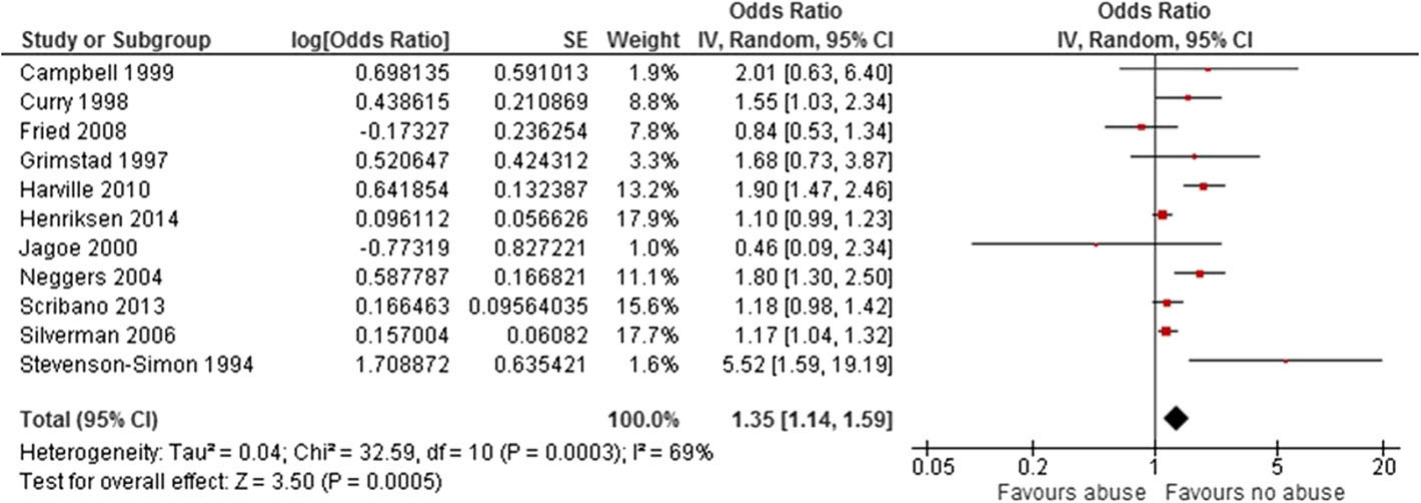
Forest plot of overall unadjusted effect estimate for low birth weight. The Forest plot of overall unadjusted effect estimate for low birth weight is shown

**Fig. 6:**
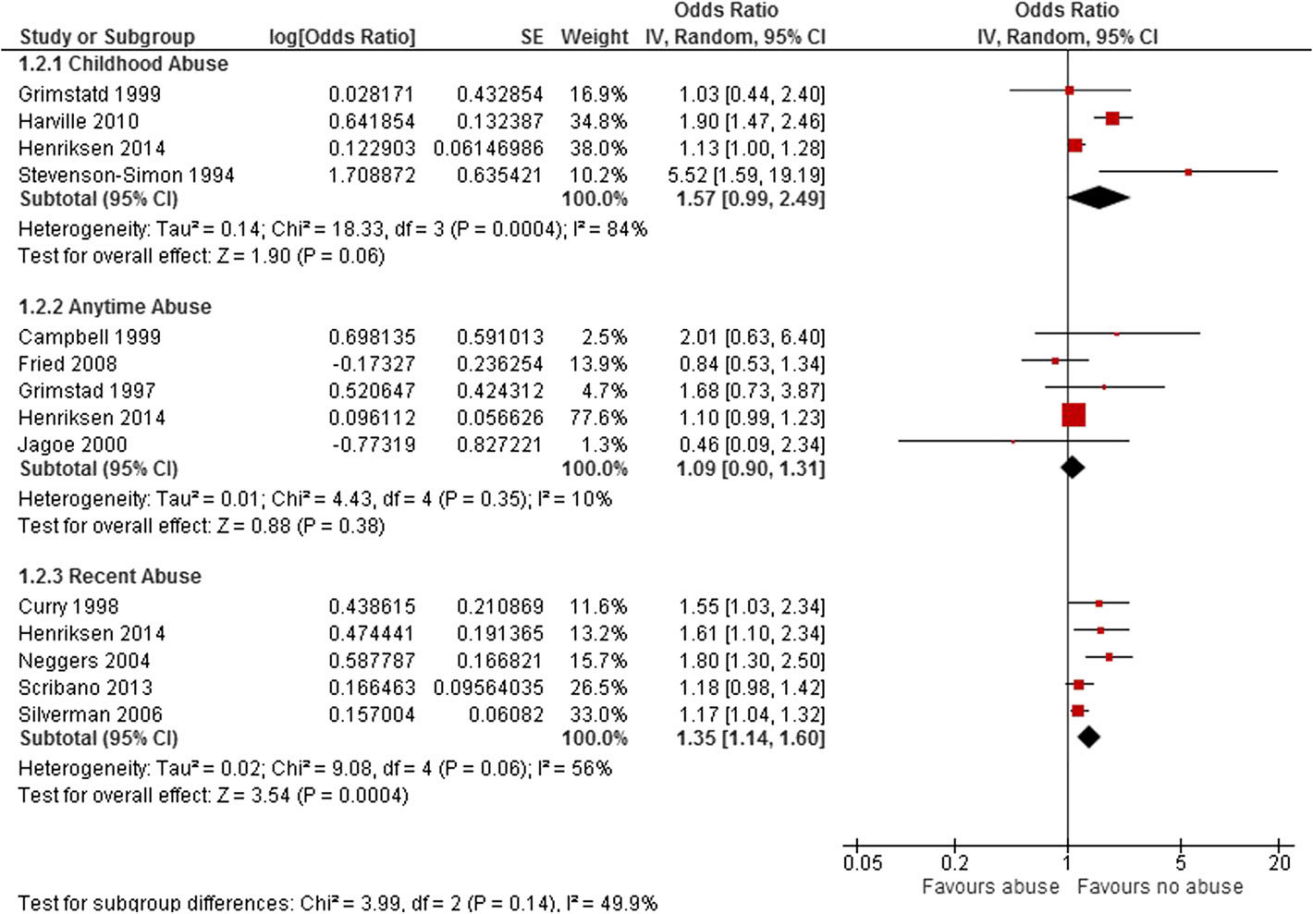
Forest plots of unadjusted effect estimate for low birth weight in subgroups of abuse time. The Forest plots of unadjusted effect estimate for low birth weight when studies categorized based on time of abuse are shown

Eleven studies examined the association between maternal histories of abuse at different points of their lives before the pregnancy of interest with LBW. Pooled OR for LBW among these studies was 1.35 (95% CI: 1.14–1.59, *p* = 0.0005) (Fig. 5). We detected a substantial level of heterogeneity in this group (I^2^ = 69%). The funnel plot for all studies included in LBW analysis was symmetric (Additional file 4). When we classified the studies by time of abuse, the pooled OR for the four studies that reported maternal history of sexual abuse in childhood was 1.57 (OR 1.57, 95% CI: 0.99–2.49, *p* = 0.06). Nevertheless, when abuse happened at any time during maternal married life (reported by five studies), odds of LBW increased by only 9% (OR 1.09, 95% CI: 0.90–1.31, *p* = 0.38). A history of recent abuse before pregnancy, reported by five studies, increased the odds of LBW by 35% (OR 1.35, 95% CI: 1.14–1.60, *p* = 0.0004) (Fig. 6). We noticed the highest degree of heterogeneity in the childhood abuse group (I^2^ = 84%). The four studies in this group that examined the association between maternal experience of sexual abuse in childhood and LBW are different in terms of sample size, study design and context of study. [32, 36, 39, 42] The study with the greatest estimated effect is a cohort study that included low income African/American women living in the United States [39], while the other studies examined women from all ethnic groups.

**Fig. 7:**
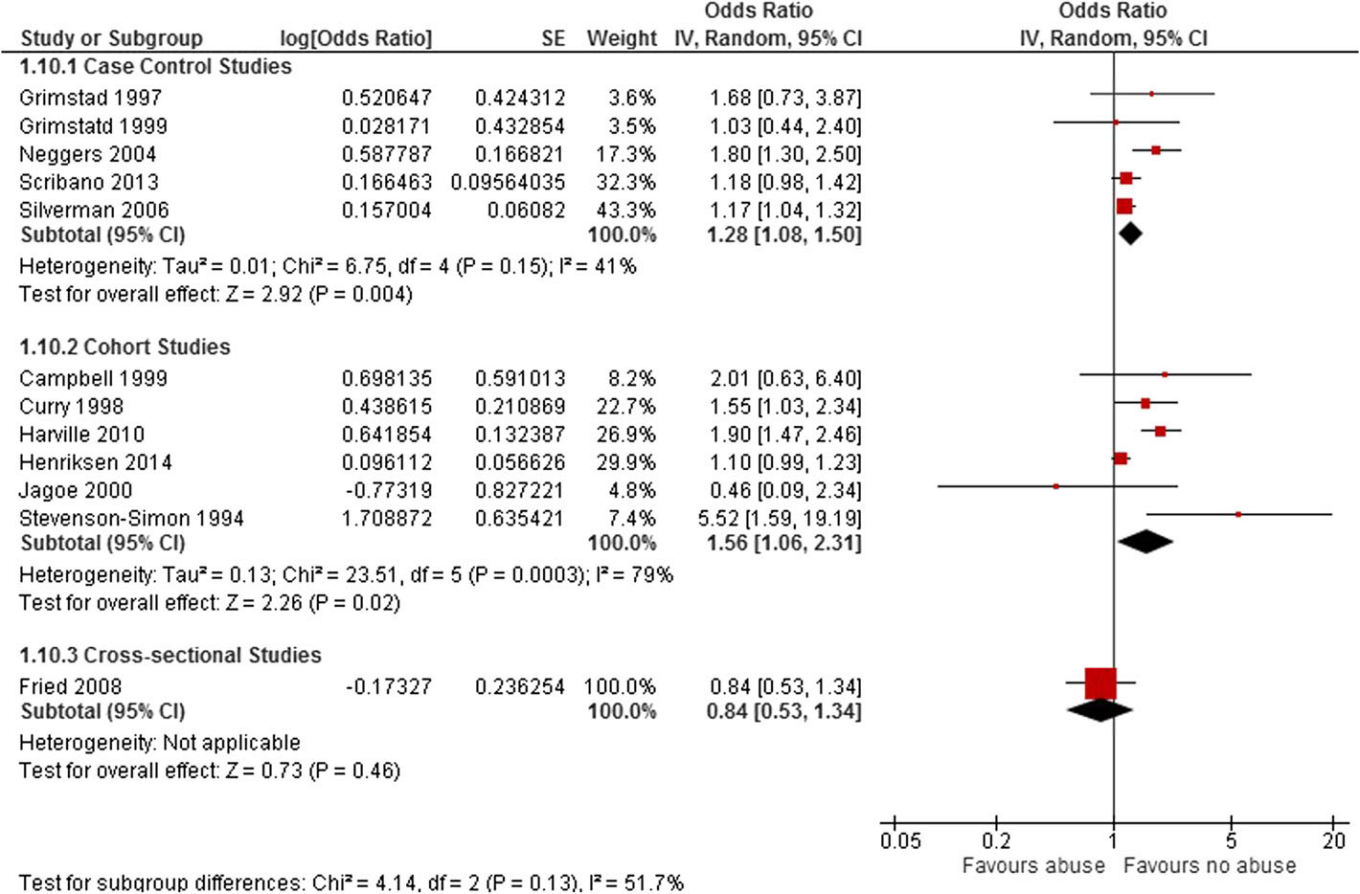
Forest plots of unadjusted effect estimate for low birth weight in subgroups of study design. The Forest plots of unadjusted effect estimate for low birth weight when studies categorized based on study design are shown

When we stratified the studies by their design, the highest estimated effect was related to the six cohort studies (OR: 1.56, 95% CI: 1.06–2.3, *p* = 0.02), while the pooled OR for the case-control group with five studies was 1.28 (95% CI: 1.08–1.50, *p* = 0.004) (Fig. 7). There was only one study with a cross-sectional design. We did not perform subgroup analysis based on study quality and context, as all the final included studies included in both PTD and LBW categories were high and moderate quality studies originating from high-income countries.

## Discussion

### Main findings

This study is the first systematic review that examined associations between maternal history of abuse occurring *before pregnancy* with PTD and LBW. Other reviews have largely explored the associations between abuses *during pregnancy* and the outcomes. Our analysis of 16 qualified articles demonstrated that women who had been abused prior to pregnancy have an increased risk of PTD and LBW compared to those who did not have the experience of abuse. In order to describe the difference in effect estimated, we stratified the included studies based on the time of exposure to abuse. From this, we learned that the subgroup of studies reporting maternal history of childhood abuse showed the highest risk for LBW among the other subgroups of recent abuse and anytime abuse in married life. Further, studies with cohort designs demonstrated the highest pooled OR for both PTD and LBW compared to case control studies.

### Strengths and limitations

The strengths of this systematic review include an extensive online search using a broad range of keywords and nine databases. We did not confine the search by study context or language. Examination of the reference lists of the included articles and related systematic reviews yielded three studies that our on-line search did not reveal. According to the assessment of risk of bias, we only included primary studies that rated as being moderate and high quality in terms of methodology. We conducted a subgroup analysis according to the time of exposure to abuse, which separates this study from other related systematic reviews examining the association between maternal history of abuse and pregnancy outcomes.

The limitations of this study suggest the results should be interpreted with caution. First, even though we only included moderate and high quality primary studies, they varied in their consideration of confounder variables to calculate adjusted OR for PTD and LBW. We also were limited by the availability of data from these studies to calculate adjusted OR controlling for the important confounders. Subsequently, we pooled unadjusted OR for this systematic review meaning that we were not able to account for other risk factors that can affect PTD and LBW. Second, we detected a substantial heterogeneity among the original studies. Because of a limited number of primary studies in each category of PTD and LBW, we did not explore all the possible sources of heterogeneity such as type of abuse, participants’ ethnic group/race, participants’ previous gestational experience, and jurisdiction and/or environment. Third, the three subgroups we created based on time of abuse are not mutually exclusive, as those who experienced childhood abuse might have been abused later. Finally, the funnel plot for the included studies in the PTD category was asymmetric indicating the possibility of publication bias. This could suggest that our computed OR from this analysis is overestimating the true odds ratio for PTD.

Further, the moderate overall effect estimated for the relationship between maternal experience of abuse before pregnancy and the two outcomes of interest in this systematic review may be partly related to the common limitations of the primary studies on abuse. Data on abuse almost always rely on the participants’ recall and perceptions. The stigmatization of abuse may prevent participants from expressing their experience. Participants may perceive abuse differently than the researcher; for instance, psychological abuse might be perceived differently across cultures. Some women may use denial as a defense mechanism to avoid painful memories of abuse. [43] The issues with detecting abuse suggest that maternal history of abuse is under reported. In addition, all the included studies in this systematic review were from high-income countries (North America, Europe, and Australia), where social support for abused women and pregnancy health services might be more available than in low-income countries. These conditions can moderate the relationship between abuse and adverse pregnancy outcomes leading to a smaller estimated effect. This is supported by the results of a recently published systematic review by Bussières et al.*,* (2015) on the effect of maternal stress on pregnancy outcomes. They reported a higher effect size for low income countries compared to that of high income countries. [44]

### Interpretation

This systematic review showed that maternal history of abuse *before pregnancy*, specifically when abuse happened in childhood, is associated with increased risk of PTD and LBW. The results contrast with the conclusion of the previous systematic reviews. Leeners et al., (2006) and Wosu et al., (2015) applying a narrative analysis, reported that the existing evidence on the link between maternal history of childhood abuse and PTD and LBW was inconsistent. [34, 35] Compared to these systematic reviews, our study had the advantage of a greater number of qualified primary studies since we searched the literature to April 2017. Compared to our results, the most recent systematic review on IPV *during pregnancy* reported higher odds for PTD (OR: 1.91, 95% CI: 1.60–2.29) and LBW (OR: 2.11, 95% CI: 1.68–2.65). For the potential mechanisms, the authors suggested that physical or sexual abuse during pregnancy are associated with placental damage, uterine contractions, premature rupture of membranes, and genitourinary infections which increase the risk PTD and LBW. [24]

One possible explanation for the observed association between maternal history of abuse *before pregnancy* and the two outcome variables is the idea that maternal experience of abuse plus other stressors during a lifetime contribute to an individual’s allostatic load. [45, 46] When the allostatic load exceeds a threshold level, the individual becomes vulnerable for diseases [47], or in the case of a pregnant woman, adverse pregnancy outcomes. [16] The findings of this systematic review can support the relationship between early life experiences, accumulation of stressors and risk of LBW and PTD. However, there are alternative explanations for the association between maternal history of abuse and the two outcomes. Having a history of abuse *before pregnancy* is related to high risk behaviors including smoking, drug abuse, and alcohol abuse. [3, 26, 30, 33, 48, 49] Moreover, abused women may not receive adequate family support or prenatal care. A maternal history of abuse can contribute to adverse pregnancy outcomes as a result of these risk factors as well. [34, 50]

## Conclusions

In this systematic review we found that women who experienced abuse *before pregnancy* had a higher risk of PTD and LBW; the highest level of risk of LBW was associated with the victims of childhood abuse. Also, the magnitude of the effect estimated by the subgroup of cohort studies for both PTD and LBW was higher than the overall effect estimated for all the included studies in each outcome category. However, respecting our study limitations, we recommend that more high quality research studies on the topic are necessary to strengthen the inference. From our examination of the existing literature, we recommend the following points to be considered when designing primary studies on this topic. First, using an appropriate study design such as a prospective cohort in which researchers have a better control on the confounding factors can lead to a more precise inference compared to other designs. Second, creating a comprehensive list of all known confounding factors related to the outcomes of interest is imperative in reducing risk of bias. Third, the complexity of detecting an abuse experience and the possibility of recall bias demand more attention to assessing history of abuse. We suggest the application of validated tools administered by trained personnel to detect the victims of abuse in research studies.

For practitioners, although the overall effect size we detected was modest, it would be prudent to more carefully examine the maternal history of abuse before pregnancy during antenatal visits in order to use this information to inform their risk assessment for adverse pregnancy outcomes. Detailed assessment of risk factors is a necessary step in order to plan for the effective management of pregnancy.

## Additional files

Additional file 1: Full search strategy, July 2015. (PDF 115 kb)

Additional file 2: Full search strategy, April 2017 (PDF 93 kb)

Additional file 3: **Figure S1.** Funnel plot for included studies in the preterm category. (JPG 40 kb)

Additional file 4: **Figure S2.** Funnel plot for all included studies in the preterm birth category. (JPG 36 kb)

## Abbreviations

LBW: Low Birth Weight
PTD: Preterm Delivery
