# Supplementary material for: Does a maternal history of abuse before pregnancy affect pregnancy outcomes? A systematic review with meta-analysis
Source: BMC Pregnancy Childbirth. 2018 Oct 16;18:404. doi: 10.1186/s12884-018-2030-8 (PMC6192330; doi:10.1186/s12884-018-2030-8)
Supplement: Supplementary file 1 — Full search strategy, July 2015. (PDF 115 kb) [file 12884_2018_2030_MOESM1_ESM.pdf]

Ovid MEDLINE(R) In-Process & Other Non-Indexed Citations, Ovid MEDLINE(R) Daily and Ovid MEDLINE(R) 1946 to Present

1. exp Premature Birth/ or exp Obstetric Labor, Premature/
2. (prematurity or pregnancy outcome* or birth outcome* or ((premature or preterm) adj3 (birth or infant* or neonat* or labo?r or delivery))).ti,ab,kf. or (preterm or prematur*).ti.
3. 1 or 2
4. Stress, Psychological/
5. stress disorders, traumatic/ or stress disorders, post-traumatic/
6. ((maternal or preconceptual or antenal or prenatal or life or chronic or lifelong or prolonged or childhood) adj3 (stress* or anxiety)).ti,ab,kf. or (lifecourse or life course).ti.
7. ((adverse adj2 experience*) or (child* adj2 neglect*)).ti,ab,kf.
8. life change events/ and stress*.mp. [mp=title, abstract, heading word, drug trade name, original title, device manufacturer, drug manufacturer, device trade name, keyword]
9. crime/ or exp crime victims/ or exp homicide/ or exp sex offenses/ or theft/ or exp violence/ or exp war crimes/ or exp war/
10. exp Battered Child Syndrome/ or exp Domestic Violence/
11. (violen* or assault* or aggression or forced sex or rape or incest or molest* or neglect* or maltreat* or mistreat* or victim* or torture*).ti,ab,kf.
12. ((verbal or physical or psychological or spous* or partner* or emotional or sexual or domestic or wife or wives or husband* or child) adj3 abus*).ti,ab,kf.
13. exp Bullying/ or (bullying or bullied or cyberbullying or cyberbullied).ti,ab,kf.
14. exp Anxiety Disorders/ or exp Depressive Disorder/
15. Poverty/ or ((social or psychological) adj3 (hardship* or stress* or disadvantage* or disparit*)).ti,ab,kf. or (economic* or socioeconomic*).ti.
16. exp Racism/ or (racism or racist* or racial).ti,ab,kf.
17. exp Disasters/ or disaster*.ti,ab,kf.
18. (anxiety or stress* or depression or depressive).ti.
19. or/4-18
20. 3 and 19
21. (associat* or correlat* or contribut* or role or risk or concomitan* or influence* or co-morbid* or relation* or expos* or predict* or mediat*).ti,ab,kf.
22. 20 and 21
23. (study or trial or measur* or incidence or prevalence or cohort or cross section* or case control or case series or retrospective or prospective or follow-up or intervention* or meta analys* or systematic review or scoping review or integrative review or panel or questionnaire* or survey*).mp. [mp=title, abstract, heading word, drug trade name, original title, device manufacturer, drug manufacturer, device trade name, keyword]
24. 22 and 23
25. limit 20 to (meta analysis or observational study or systematic reviews or review)
26. limit 24 to human
27. limit 24 to animal
28. 26 and 27
29. 26 or 27
30. 24 not 29
31. 25 or 26 or 28 or 30

Ovid EMBASE, 1974-Current

1. exp *Prematurity/ or exp *Premature labor/
2. (prematurity or pregnancy outcome* or birth outcome* or ((premature or preterm) adj3 (birth or infant* or neonat* or labo?r or deliver*))).ti,ab. or (preterm or prematur*).ti.
3. 1 or 2
4. exp maternal stress/ or exp *mental stress/ or exp *chronic stress/ or exp *school stress/ or exp *early life stress/ or exp *social stress/ or exp *role stress/ or exp *critical incident stress/ or exp *stress/ or exp *home stress/ or exp *interpersonal stress/ or exp *emotional stress/ or exp *family stress/ or exp *life stress/ or exp *posttraumatic stress disorder/
5. ((maternal or preconceptual or antenal or prenatal or life or chronic or lifelong or prolonged or childhood or lifecourse) adj3 (stress* or anxiety)).ti,ab.
6. ((adverse adj2 experience*) or (child* adj2 neglect*)).ti,ab.
7. life event/ and stress*.mp.
8. exp crime/ or exp crime victim/ or exp violence/ or exp war/ or exp torture survivor/ or exp torture/ or exp domestic violence/
9. (violen* or assault* or aggression or forced sex or rape or incest or molest* or neglect* or maltreat* or mistreat* or victim* or torture*).ti,ab.
10. ((verbal or physical or psychological or spous* or partner* or emotional or sexual or domestic or wife or wives or husband* or child) adj3 abus*).ti,ab.
11. exp Bullying/ or (bullying or bullied or cyberbullying or cyberbullied).ti,ab.
12. exp *Anxiety Disorders/ or exp *Depression/
13. Poverty/ or Socioeconomics/ or ((social or psychological) adj3 (hardship* or stress* or disadvantage* or disparit*)).ti,ab. or (economic* or socioeconomic*).ti.
14. exp Racism/ or (racism or racist* or ((racial or ethnic*) adj2 (discriminat* or prejudic*))).ti,ab.
15. exp Disaster/ or disaster*.ti,ab.
16. (anxiety or stress* or depression or depressive).ti.
17. or/4-16
18. 3 and 17
19. (associat* or correlat* or contribut* or role or risk or concomitan* or influence* or co-morbid* or relation* or expos* or predict* or mediat*).ti,ab.
20. 18 and 19
21. (study or trial or measur* or incidence or prevalence or cohort or cross section* or case control or case series or retrospective or prospective or follow-up or intervention* or meta analys* or systematic review or scoping review or integrative review or questionnaire* or survey* or analys* or statisti* or population research or review).mp.
22. 20 and 21
23. limit 20 to "review"
24. 22 or 23

Ovid PsycInfo, 1806-Current

1. exp Premature Birth/
2. (prematurity or pregnancy outcome* or birth outcome* or ((premature or preterm) adj3 (birth or infant* or neonat* or labo?r or deliver*))).ti,ab,id.

3. 1 or 2
4. exp stress/ or exp anxiety/ or exp disasters/ or family crises/ or posttraumatic stress disorder/ or acute stress disorder/ or emotional trauma/
5. ((maternal or preconceptual or antenal or prenatal or life or chronic or lifelong or prolonged or childhood or lifecourse) adj3 (stress* or anxiety)).ti,ab,id.
6. ((adverse adj2 experience*) or (child* adj2 neglect*)).ti,ab,id.
7. (exp Life Changes/ or exp Life Experiences/) and stress*.mp.
8. exp crime/ or exp crime victims/ or exp violence/ or exposure to violence/ or hate crimes/ or exp partner abuse/ or physical abuse/ or torture/ or exp war/
9. child neglect/ or ((infant* or child*) adj2 neglect*).ti,ab,id.
10. (violen* or assault* or aggression or forced sex or rape or incest or molest* or neglect* or maltreat* or mistreat* or victim* or torture*).ti,ab,id.
11. ((verbal or physical or psychological or spous* or partner* or emotional or sexual or domestic or wife or wives or husband* or child) adj3 abus*).ti,ab,id.
12. exp Bullying/ or (bullying or bullied or cyberbullying or cyberbullied).ti,ab,id.
13. exp Anxiety Disorders/ or exp Major Depression/
14. Poverty/ or ((social or psychological) adj3 (hardship* or stress* or disadvantage* or disparit*)).ti,ab,id. or (socioeconomic* or economic*).ti.
15. social discrimination/ or "race and ethnic discrimination"/ or racism/ or (racism or racist* or ((racial or ethnic*) adj2 (discriminat* or prejudic*))).ti,ab,id.
16. exp Disasters/ or disaster*.ti,ab,id.
17. (anxiety or stress* or depression or depressive).ti.
18. or/4-17
19. 3 and 18
20. (associat* or correlat* or contribut* or role or risk or concomitan* or influence* or co-morbid* or relation* or expos* or predict* or mediat*).ti,ab.
21. 19 and 20
22. (study or trial or incidence or prevalence or cohort or cross section* or case control or case series or retrospective or prospective or follow-up or intervention* or meta analys* or systematic review or scoping review or integrative review or questionnaire* or survey* or review).mp.
23. 21 and 22
24. limit 21 to ("0400 empirical study" or "0430 followup study" or "0450 longitudinal study" or "0451 prospective study" or "0453 retrospective study" or "0830 systematic review" or 1200 meta analysis or 1800 quantitative study)
25. 23 or 24

Ovid EBM Reviews: Cochrane Database of Systematic Reviews

1. (prematurity or pregnancy outcome* or birth outcome* or ((premature or preterm) adj3 (birth or infant* or neonat* or labo?r))).ti,ab,kw.
2. ((maternal or preconceptual or antenal or prenatal or life or chronic or lifelong or prolonged or childhood or lifecourse) adj3 (stress* or anxiety)).ti,ab,kw.
3. ((adverse adj2 experience*) or (child* adj2 neglect*)).ti,ab,kw.
4. ((infant* or child*) adj2 neglect*).ti,ab,kw.
5. (violen* or assault* or aggression or forced sex or rape or incest or molest* or neglect* or maltreat* or mistreat* or victim* or torture*).ti,ab,kw.

6. ((verbal or physical or psychological or spous* or partner* or emotional or sexual or domestic or wife or wives or husband* or child) adj3 abus*).ti,ab,kw.
7. (bullying or bullied or cyberbullying or cyberbullied).ti,ab,kw.
8. ((social or psychological) adj3 (hardship* or stress* or disadvantage* or disparit*)).ti,ab,kw. or (socioeconomic* or economic*).ti.
9. (racism or racist* or ((racial or ethnic*) adj2 (discriminat* or prejudic*))).ti,ab,kw.
10. disaster*.ti,ab,kw.
11. (anxiety or stress* or depression or depressive).ti,kw.
12. or/2-11
13. 1 and 12
14. (associat* or correlat* or contribut* or role or risk or concomitan* or influence* or co-morbid* or relation* or expos* or predict* or mediat*).ti,ab,kw.
15. 13 and 14

Ovid EBM Reviews: Cochrane Central Register of Controlled Trials

1. exp Premature Birth/ or exp Obstetric Labor, Premature/
2. (prematurity or pregnancy outcome* or birth outcome* or ((premature or preterm) adj3 (birth or infant* or neonat* or labo?r or deliver*))).ti,ab.
3. 1 or 2
4. Stress, Psychological/
5. stress disorders, traumatic/ or stress disorders, post-traumatic/
6. ((maternal or preconceptual or antenal or prenatal or life or chronic or lifelong or prolonged or childhood or lifecourse) adj3 (stress* or anxiety)).ti,ab.
7. ((adverse adj2 experience*) or (child* adj2 neglect*)).ti,ab.
8. life change events/ and stress*.mp.
9. crime/ or exp crime victims/ or exp homicide/ or exp sex offenses/ or theft/ or exp violence/ or exp war crimes/ or exp war/
10. exp Battered Child Syndrome/ or exp Domestic Violence/
11. (violen* or assault* or aggression or forced sex or rape or incest or molest* or neglect* or maltreat* or mistreat* or victim* or torture*).ti,ab.
12. ((verbal or physical or psychological or spous* or partner* or emotional or sexual or domestic or wife or wives or husband* or child) adj3 abus*).ti,ab.
13. exp Bullying/ or (bullying or bullied or cyberbullying or cyberbullied).ti,ab.
14. exp Anxiety Disorders/ or exp Depressive Disorder/
15. Poverty/ or ((social or psychological) adj3 (hardship* or stress* or disadvantage* or disparit*)).ti,ab. or (economic* or socioeconomic*).ti.
16. exp Racism/ or (racism or racist* or ((racial or ethnic*) adj2 (discriminat* or prejudic*))).ti,ab.
17. exp Disasters/ or disaster*.ti,ab.
18. (anxiety or stress* or depression or depressive).ti.
19. or/4-18
20. 3 and 19
21. (associat* or correlat* or contribut* or role or risk or concomitan* or influence* or co-morbid* or relation* or expos* or predict* or mediat*).ti,ab.
22. 20 and 21

Scopus, 1960-Current

(TITLE-ABS-KEY((verbal W/3 abus*) OR (physical W/3 abus*) OR (psychological W/3 abus*) OR (adverse W/3 experience*) OR violen* OR assault* OR aggression OR "forced sex" OR rape OR incest OR molest* OR neglect* OR maltreat* OR mistreat* OR victim* OR (spous* W/3 abus*) OR (wife W/3 abus*) OR (wives W/3 abus*) OR (partner W/3 abus*) OR (husband W/3 abus*) OR (child W/3 abus*) OR (sexual W/3 abus*) OR (domestic W/3 abus*) OR bullying OR bullied OR cyberbullying OR cyberbullied OR torture* or poverty or ((socioeconomic or economic or social or psychological) w/3 (hardship* or stress* or disadvantage* or disparit*))) OR TITLE-ABS-KEY (((social or psychological) w/3 (hardship* or stress* or disadvantage* or disparit*)) or racism or racist* or ((racial or ethnic*) w/2 (discriminat* or prejudic*)) or disaster*) OR TI(socioeconomic* or economic*)) AND TITLE-ABS-KEY((preterm w/2 (birth or labor or labour)) or (premature w/2 (birth or labor or labour or deliver*)) or prematurity)

Proquest PILOTS (Published International Literature on Traumatic Stress)

(preterm or premature) w/3 (birth or labor or labour) or prematurity

Web of Science Core Collection, 1900-Current

TS= ((premature or preterm) near/3 (birth or infant* or neonat* or labor or labour or deliver*)) AND (TS=((maternal or preconceptual or antenal or prenatal or life or chronic or lifelong or prolonged or childhood or lifecourse) near/3 (stress* or anxiety) or (adverse near/2 experience*) or ((child* or infant*) near/2 neglect*) or ((verbal or physical or psychological or spous* or partner* or emotional or sexual or domestic or wife or wives or husband* or child) near/3 abus*) or violen* or assault* or aggression or forced sex or rape or incest or molest* or neglect* or maltreat* or mistreat* or victim* or torture* or bullying or bullied or cyberbullying or cyberbullied or ((socioeconomic or economic or social or psychological) near/3 (hardship* or stress* or disadvantage*)) or racism or racist* or ((racial or ethnic*) near/2 (discriminat* or prejudic*)) or disaster*) or TI=(anxiety or stress* or depression or depressive)) AND TS=(associat* or correlat* or contribut* or role or risk or concomitan* or influence* or co-morbid* or relation* or expos* or predict* or mediat*) AND TS=(study or trial or incidence or prevalence or cohort or cross section* or retrospective or prospective or follow-up or intervention* or meta analys* or systematic review or scoping review or integrative review or questionnaire* or survey* or review)

EBSCO CINAHL PLUS with Full-text, 1937-Current

S1 ((MH "Childbirth, Premature") OR (MH "Labor, Premature")) OR ((premature or preterm) n3 (birth or infant* or neonat* or labor or labour or deliver*))
S2 (MH "Life Change Events+") AND (MH "Stress, Psychological+") OR (MH "Crime+") OR (MH "Crime Victims") OR (MH "Verbal Abuse") OR (MH "Torture") OR (MH "Bullying") OR (MH "Stress Disorders, Post-Traumatic+") OR (MH "War+") OR (MH "Domestic Violence+")
S3 ((adverse N3 experience*) or violen* or torture* or assault* or aggression or forced sex or rape or incest or molest* or neglect* or maltreat* or mistreat* or victim*)) OR ((verbal or physical or psychological or spous* or elder* or partner* or emotional or sexual or domestic or wife or wives or husband* or child) N3 abus*) OR (bullying or bullied or cyberbullying or cyberbullied)
S4 (child* or infant*) n2 neglect*

S5 ((MH "Poverty+") OR (MH "Socioeconomic Factors+")) OR (poverty or (social or psychological) w3 (hardship* or stress* or disadvantage* or disparit*)) OR TI (economic* or socioeconomic*)
S6 racism or racist* or (racial or ethnic*) w2 (discriminat* or prejudic*)
S7 (MH "Race Factors")
S8 (MH "Anxiety Disorders+") OR (MH "Depression+")
S9 ((MH "Anxiety Disorders+") OR (MH "Depression+")) OR TI (anxiety or depress or depressive or stress)
S10 S2 OR S3 OR S4 OR S5 OR S6 OR S7 OR S8 OR S9
S11 S1 AND S10
