# Supplementary material for: Does a maternal history of abuse before pregnancy affect pregnancy outcomes? A systematic review with meta-analysis
Source: BMC Pregnancy Childbirth. 2018 Oct 16;18:404. doi: 10.1186/s12884-018-2030-8 (PMC6192330; doi:10.1186/s12884-018-2030-8)
Supplement: Supplementary file 2 — Full search strategy, April 2017 (PDF 93 kb) [file 12884_2018_2030_MOESM2_ESM.pdf]

Ovid MEDLINE(R) Epub Ahead of Print, In-Process & Other Non-Indexed Citations, Ovid MEDLINE(R) Daily and Ovid MEDLINE(R) <1946 to Present>

1 exp Premature Birth/ or exp Obstetric Labor, Premature/
2 (((premature or preterm) adj3 (birth or infant* or neonat* or labo?r or delivery)) or prematurity).ti,ab,kf.
3 exp Infant, Low Birth Weight/
4 (birthweight or birth weight).ti,ab,kf.
5 Fetal Growth Retardation/ or (small for gestational age or small for date).ti,ab,kf. or ((fetal growth or intrauterine growth) adj2 (retard* or restrict*)).ti,ab,kf.
6 or/1-5
7 exp domestic violence/ or spouse abuse/ or intimate partner violence/ or physical abuse/
8 (violen* or assault* or aggression or forced sex or rape or incest or molest* or neglect* or maltreat* or mistreat* or torture* or victim* or battered wom#n).ti,ab,kf.
9 ((verbal or physical or psychological or spous* or partner* or emotional or sexual or domestic or wife or wives or husband* or child*) adj3 abus*).ti,ab,kf.
10 or/7-9
11 6 and 10
12 remove duplicates from 11

Ovid EMBASE, 1974-Current

1 exp Prematurity/ or exp Premature labor/
2 (((premature or preterm) adj3 (birth or infant* or neonat* or labo?r or deliver*)) or prematurity).ti,ab,kw.
3 exp low birth weight/
4 (birth weight or birthweight).ti,ab,kw.
5 intrauterine growth retardation/ or exp small for date infant/ or (small for gestational age or small for date).ti,ab,kw. or ((fetal growth or intrauterine growth) adj2 (retard* or restrict*)).ti,ab,kw.
6 or/1-5
7 exp domestic violence/
8 (violen* or assault* or aggression or forced sex or rape or incest or molest* or neglect* or maltreat* or mistreat* or victim* or torture*).ti,ab,kw.
9 ((verbal or physical or psychological or spous* or partner* or emotional or sexual or domestic or wife or wives or husband* or child*) adj3 abus*).ti,ab,kw.
10 or/7-9
11 6 and 10
12 remove duplicates from 11

Wiley COCHRANE Database of Systematic Reviews and Central Register of Controlled Trials

#1 [mh "premature birth"] or [mh "obstetric labor, premature"] or [mh "infant, low birth weight"] or [mh "fetal growth retardation"]
#2 ((premature or preterm) near/3 (birth or infant* or neonat* or labor or labour or delivery)):ti,ab,kw
#3 (birthweight or "birth weight" or "small for gestational age" or "small for date"):ti,ab,kw
#4 (("fetal growth" or "intrauterine growth") near/2 (retard* or restrict*)):ti,ab,kw
#5 #1 OR #2 OR #3 OR #4

#6 [mh "domestic violence"] OR [mh "spouse abuse"] OR [mh "intimate partner violence"] OR [mh "physical abuse"]
#7 (violen* or assault* or aggression or "forced sex" or rape or incest or molest* or neglect* or maltreat* or mistreat* or torture* or victim* or "battered women" or "battered woman"):ti,ab,kw
#8 ((verbal or physical or psychological or spous* or partner* or emotional or sexual or domestic or wife or wives or husband* or child*) near/3 abus*):ti,ab,kw
#9 #6 OR #7 OR #8
#10 #5 AND #9

Scopus, 1960-Current

(TITLE-ABS-KEY(((premature or preterm) pre/3 (birth or infant* or neonat* or labor or labour or deliver*)) or prematurity OR "small for gestational age" or "small for date" or (("fetal growth" or "intrauterine growth") w/2 (restrict* or retard*))) AND TITLE(violen* or assault* or aggression or "forced sex" or rape or incest or molest* or neglect* or maltreat* or mistreat* or torture* or victim* or "battered women" or "battered woman" OR ((verbal or physical or psychological or spous* or partner* or emotional or sexual or domestic or wife or wives or husband* or child*) w/3 abus*))) OR (TITLE(((premature or preterm) pre/3 (birth or infant* or neonat* or labor or labour or deliver*)) or prematurity OR "small for gestational age" or "small for date" or (("fetal growth" or "intrauterine growth") w/2 (restrict* or retard*))) AND TITLE-ABS-KEY(violen* or assault* or aggression or "forced sex" or rape or incest or molest* or neglect* or maltreat* or mistreat* or torture* or victim* or "battered women" or "battered woman" OR ((verbal or physical or psychological or spous* or partner* or emotional or sexual or domestic or wife or wives or husband* or child*) w/3 abus*)))

Web of Science Core Collection, 1900-Current

(premature or preterm) near/3 (birth or infant* or neonat* or labor or labour or deliver*) or prematurity OR "small for gestational age" or "small for date" or ("fetal growth" or "intrauterine growth") near/2 (restrict* or retard*)
AND
violen* or assault* or aggression or "forced sex" or rape or incest or molest* or neglect* or maltreat* or mistreat* or torture* or victim* or "battered women" or "battered woman" OR (verbal or physical or psychological or spous* or partner* or emotional or sexual or domestic or wife or wives or husband* or child*) near/3 abus*

EBSCO CINAHL Plus with Full-text, 1937-Current

((MH "Childbirth, Premature") OR (MH "Infant, Low Birth Weight+") OR (MH "Infant, Premature") OR (MH "Labor, Premature") OR (MH "Infant, Small for Gestational Age") OR (MH "Fetal Growth Retardation")) OR ((premature or preterm) w3 (birth or infant* or neonat* or labor or labour or deliver*) or prematurity) OR ("small for gestational age" or "small for date" or ("fetal growth" or "intrauterine growth") n2 (restrict* or retard*))
AND
((MH "Domestic Violence+") OR (MH "Torture") OR (MH "Verbal Abuse")) OR (violen* or assault* or aggression or "forced sex" or rape or incest or molest* or neglect* or maltreat* or mistreat* or torture* or victim* or "battered women" or "battered woman") OR ((verbal or physical or psychological or spous* or partner* or emotional or sexual or domestic or wife or wives or husband* or child*) n3 abus*)

EBSCO Violence & Abuse Abstracts, 1971-Current

((premature or preterm) w3 (birth or infant* or neonat* or labor or labour or deliver*) or prematurity) OR ("small for gestational age" or "small for date" or ("fetal growth" or "intrauterine growth") n2 (restrict* or retard*))
AND
(violen* or assault* or aggression or "forced sex" or rape or incest or molest* or neglect* or maltreat* or mistreat* or torture* or victim* or "battered women" or "battered woman") OR ((verbal or physical or psychological or spous* or partner* or emotional or sexual or domestic or wife or wives or husband* or child*) n3 abus*)

ProQuest PILOTS (Published International Literature on Traumatic Stress)

((premature OR preterm) PRE/3 (birth OR infant* OR neonat* OR labor OR labour OR deliver*) OR prematurity) OR ("small for gestational age" OR "small for date" OR ("fetal growth" OR "intrauterine growth") NEAR/2 (restrict* OR retard*))
AND
(violen* OR assault* OR aggression OR "forced sex" OR rape OR incest OR molest* OR neglect* OR maltreat* OR mistreat* OR torture* OR victim* OR "battered women" OR "battered woman") OR ((verbal OR physical OR psychological OR spous* OR partner* OR emotional OR sexual OR domestic OR wife OR wives OR husband* OR child*) NEAR/3 abus*)

ProQuest Dissertations & Theses Global, 1861-Current

((premature OR preterm) PRE/3 (birth OR infant* OR neonat* OR labor OR labour OR deliver*) OR prematurity) OR ("small for gestational age" OR "small for date" OR ("fetal growth" OR "intrauterine growth") NEAR/2 (restrict* OR retard*))
AND
(violen* OR assault* OR aggression OR "forced sex" OR rape OR incest OR molest* OR neglect* OR maltreat* OR mistreat* OR torture* OR victim* OR "battered women" OR "battered woman") OR ((verbal OR physical OR psychological OR spous* OR partner* OR emotional OR sexual OR domestic OR wife OR wives OR husband* OR child*) NEAR/3 abus*)
