# Supplementary figures and images for: Does a maternal history of abuse before pregnancy affect pregnancy outcomes? A systematic review with meta-analysis
Source: BMC Pregnancy Childbirth. 2018 Oct 16;18:404. doi: 10.1186/s12884-018-2030-8 (PMC6192330; doi:10.1186/s12884-018-2030-8)

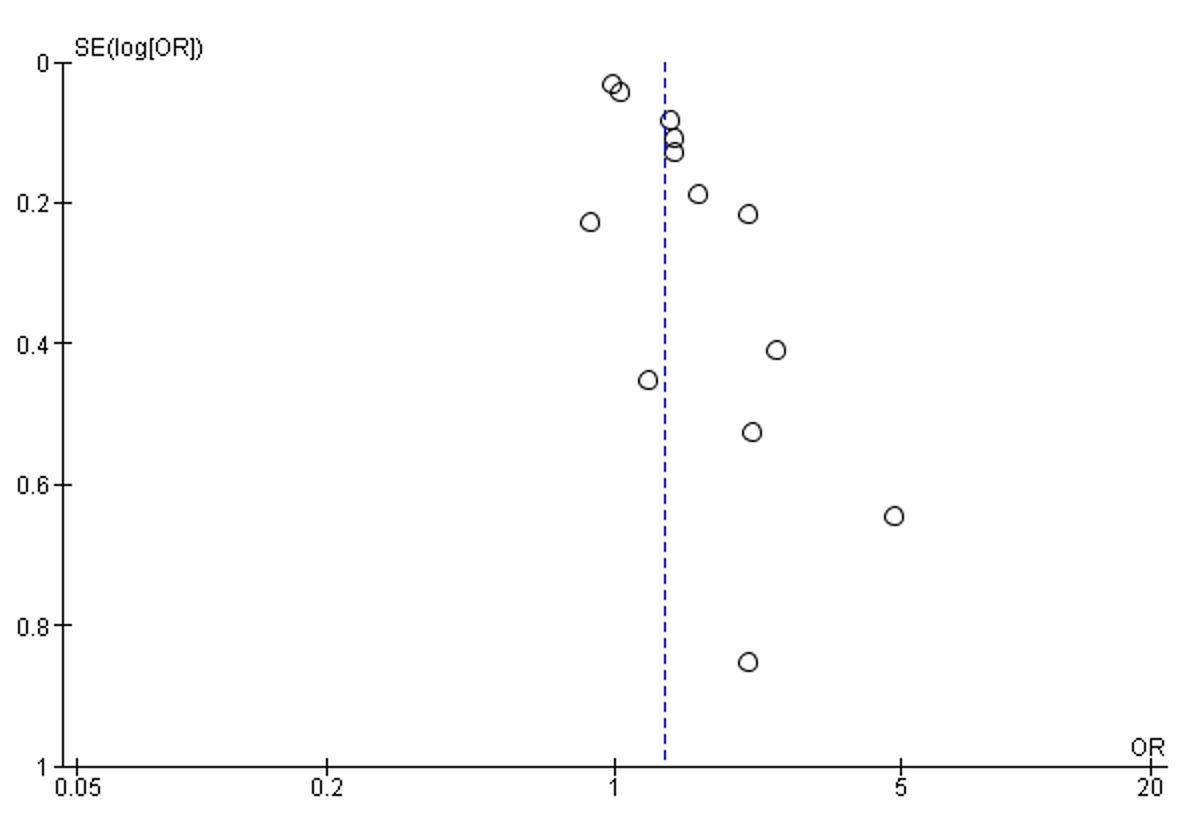

Supplement: Supplementary file 3 — Figure S1. Funnel plot for included studies in the preterm category. (JPG 40 kb) [file 12884_2018_2030_MOESM3_ESM.jpg]

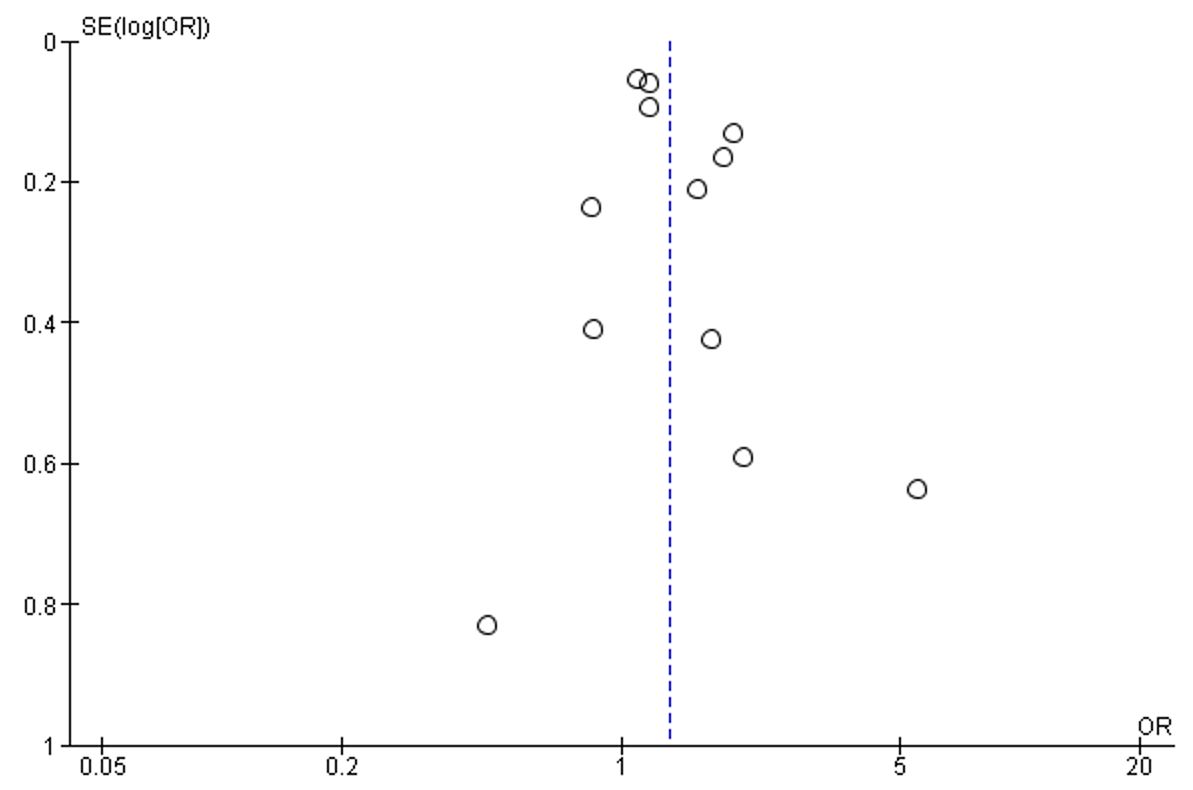

Supplement: Supplementary file 4 — Figure S2. Funnel plot for all included studies in the preterm birth category. (JPG 36 kb) [file 12884_2018_2030_MOESM4_ESM.jpg]
